# 861. Development and Clinical Validation of a Custom Modified Two-Tier Testing Algorithm for Serodiagnosis of Lyme Disease

**DOI:** 10.1093/ofid/ofad500.906

**Published:** 2023-11-27

**Authors:** Michelle A Gaylord, Danielle Baranova, Michael W Pride, Raphael Simon, Annaliesa S Anderson, Warren Vincent Kalina

**Affiliations:** Vaccine Research and Development, Pfizer, Pearl River, New York; Pfizer Inc., Pearl river, New York; Pfizer Vaccines, Pearl River, New York; Pfizer, New York, New York; Pfizer, New York, New York; Pfizer Vaccine Research and Development, Pearl River, NY

## Abstract

**Background:**

Diagnosis of Lyme Disease has relied on measuring the serological response to *Borrelia sp*. Over the years the rigor of serological diagnosis has evolved to meet the needs of the clinic and vaccine manufacturers, where the latter require especially sensitive and specific tests to measure efficacy. Likewise, current US guidelines recommend a two-tier testing approach to diagnose Lyme Disease. Among a constellation of commercially available *in vitro* diagnostics, two highly sensitive immunoassays with *Borrelia sp.* antigenic diversity were characterized for utility as a custom Modified Two-Tier Testing (MTTT) algorithm. This custom algorithm consists of a first tier assay from Bio-Rad and a second tier assay from Zeus Scientific, and was developed to capture cases of Lyme Disease.

**Methods:**

The BioPlex 2200 Lyme Total assay from BioRad and VlsE1/pepC10 IgG/IgM ELISA from Zeus Scientific were evaluated as the first and second tier assays in the custom MTTT, respectively. The MTTT was clinically assessed for diagnostic accuracy and compared to the highly characterized CDC Pre-Marketing Panel, which is composed of samples from US donors diagnosed with Lyme Disease, “look-alike” diseases or healthy individuals. Other clinically relevant sample populations were benchmarked off the predicate FDA-approved MTTT, also from Zeus Scientific.

**Results:**

The custom MTTT, described here, was more clinically specific than either test alone and was more clinically sensitive than the only other FDA-approved MTTT. When matched against the FDA-approved predicate method, sensitivity and specificity was 100%. The custom MTTT also detected fewer “look-alike” disease samples than either test alone, or the FDA-approved MTTT. Furthermore, detection of early Lyme Disease in the CDC Pre-Marketing panel was 80% sensitive. In addition, the custom MTTT identified Lyme disease in 98.6% of Lyme Disease samples collected from samples that originated in Europe.

**Conclusion:**

The clinically validated custom MTTT method, described here, has utility as a Lyme Disease diagnostic and is a highly specific tool for assessing efficacy of Lyme Disease vaccines currently in development.

**Disclosures:**

**Michelle A. Gaylord, PhD**, Pfizer Inc.: Stocks/Bonds **Danielle Baranova, PhD**, Pfizer Inc: Stocks/Bonds **Michael W. Pride, PhD**, Pfizer: Stocks/Bonds **Raphael Simon, PhD**, Pfizer: Stocks/Bonds **Annaliesa S. Anderson, PhD**, Pfizer: Employee|Pfizer: Stocks/Bonds **Warren Vincent. Kalina, PhD**, Pfizer: Stocks/Bonds

